# Hearing aids fitting process in users that are seen in a federal public institution: part I -results and implications with the amplification device

**DOI:** 10.1016/S1808-8694(15)31170-8

**Published:** 2015-10-19

**Authors:** Carine Dias de Freitas, Maristela Julio Costa

**Affiliations:** 1 M.Sc. in Human Communications Disorders - Federal University of Santa Maria - UFSM/RS. Speech and Hearing Therapist. Substitute Professor - Speech and Hearing Therapy Course - Federal University of Santa Maria - UFSM/RS; 2 PhD in Human Communications Disorders /Speech and Hearing Therapy - Federal University of São Paulo - UNIFESP/SP. Speech and Hearing Therapist. Adjunct Professor - Speech and Hearing Therapy Course - Federal Dissertation entitled: “Resultados e implicações do processo de adaptação de próteses auditivas em usuários atendidos em uma instituição pública federal” apresentada ao Curso de Mestrado do Programa de Pós-Graduação em Distúrbios da Comunicação Humana, Área de Concentração em Audiologia, da Universidade Federal de Santa Maria (UFSM, RS), como requisito parcial para obtenção do grau de Mestre em Distúrbios da Comunicação Humana, 2006. Apresentado como Atualização no XXI Encontro Internacional de Audiologia, realizado em Bauru - SP, 2006

**Keywords:** hearing loss, hearing aid, results

## Abstract

Hearing aid fitting involves numerous issues that must be investigated - from the device supply all the way to results achieved with it in order to improve its results in planning Auditory Health Care. **Aims**: to verify difficulties related to device use, batteries and ear molds handling, the very characteristics of sound amplification, and to discuss factors that impact audiological rehabilitation in this group of patients.

**Materials and Methods:**

31 individuals, from 12 to 77 years age, with bilaterally symmetrical hearing losses, sensorineural or mixed, from moderate to moderately severe degrees and hearing aid users of digital or hybrid hearing aids. We carried out an interview approaching topics related to device use, battery handling, ear molds and hearing aids, as well as sound amplification characteristics.

**Results:**

12.90%, 58.06% and 67.74% of the interviewees presented difficulties related to battery, ear molds or capsules and amplification characteristics, respectively.

**Conclusion:**

the majority of the individuals presented some kind of complaint related to hearing aids characteristics, and difficulties related to users' expectations, communication skills and monetary cost and the Municipal Health Care Network, which all interfered in the audiological rehabilitation.

## INTRODUCTION

How one defines hearing loss varies according to different educational, social and audiological aspects, as well as the physician involved. Notwithstanding, it is a highly disabling disorder, considering its impact on communication, cognitive, psychosocial, oral and written language.

With the development of medicine and technological progress, it became possible to improve quality of life, including people with hearing loss. Thus, sound amplification systems have been developed and are constantly improved in order to minimize the limiting effects caused by such disability^1^.

Sound amplification is not restricted only to help with warning signs, but mainly to make audible the sounds of speech, a tool used to facilitate education and the psychosocial and intellectual development of the hearing impaired^2^, that is why it is necessary to survey the hearing loss situation in our country, especially in relation to hearing aids supply and the user's satisfaction after receiving it, this could help better plan measures aiming at Auditory Health Care^3^.

In Brazil, according to estimates from the World Health Organization, there are about 2.25 million people with hearing impairment. In order to guarantee specialized care to everyone, the Ministry of Health created the National Policy of Hearing Health Care. Two ordinances associated with the Secretary of Health Care standardized this new policy: ordinance 587, from October 07, 2004, and ordinance 589, October 08, 20043.

According to the strategies of this new policy, the audiologic rehabilitation process involves a much broader work, aiming at providing continuous flow hearing aids with medical and audiologic follow up, both for adjustments and for periodic inspections of the device and the benefit brought about by it; and whenever necessary, speech and hearing therapy and social and psychological care.

Although the new ordinances were only created in 2004, this issue of an Auditory Health Care had already been discussed. Thus, based on Ordinance 432 from the Ministry of Health, the State Health Secretary of Rio Grande do Sul, in order to establish joint actions to provide hearing aids, established a consortium with the Federal University of Santa Maria in order to care for the users of the Sistema Único de Saúde (Brazilian Public Health Care System), for people with hearing impairment, signed on 12/29/2000 and published in the Federal Gazette on 02/08/2001, which was put into effect by 2005.

In the area of audiology, many surveys are being carried out with users of hearing aids, however, no studies were found involving users of the Public Health Care System in Brazil. Thus, the first part of this paper aimed at:

1. Checking the difficulties associated with the use and handling of batteries and ear molds, and sound amplification characteristics;
2. Discuss the many aspects involved in the hearing aid fitting processes, education and follow up of this study group.

## MATERIALS AND METHODS

This study was carried out at the Audiology Outpatient Ward of the Speech and Hearing Care Center (SAF) of the Federal University of Santa Maria (UFSM), from July of 2004 to July of 2005, after being approved by the Ethics Committee, under protocol # 112/2004 - UFSM. We evaluated only those individuals who agreed to participate in the project, after they were fully educated about the objective and methodology of our study and, after they signed the Informed Consent Form.

The individuals evaluated in this study were fitted in the Hearing Aid Laboratory of the Federal University of Santa Maria by means of a consortium established between the State Health Secretary and the Federal University of Santa Maria.

To form the group, we established the following criteria:

– Age equal to or above 12 years;
– Hearing aid in both ears;
– Patients of mild to moderately severe sensorineural or mixed hearing loss4;
– Symmetrical hearing loss, considering a maximum difference of 10dB between the same frequencies in both ears;
– Minimum time of three months of hearing aid use^5^,^6^,^7^;
– Users of hearing aids with digital signal processing or computer programmable analogue signal.

Coming from different locations of the Midwest of Rio Grande do Sul State, the individuals in this investigation, part of the low income population, were selected based on personal and audiologic data which were previously surveyed at the time of hearing aid selection and fitting. Of the 224 individuals fitted from April of 2003 to July of 2005, according to the study criteria, 37 individuals were selected.

Most of the appointments were scheduled through the Municipal Health Secretariat. Of the 76 appointments scheduled, only 31 individuals came to 46 of them.

Thus, the study involved 31 individuals, 51.61% (N = 16) males and 48.39% (N = 15) females, at an age range between 12 and 77 (mean value of 38.87 years), all with symmetrical bilateral sensorineural or mixed hearing loss, of moderate to moderately-severe level, users of miniretroauricular and intracanal hearing devices, digital or computer-programmable analogue type.

We established a minimum time of three months of

**ATTACHMENT I**

FEDERAL UNIVERSITY OF SANTA MARIA

HEALTH SCIENCES CENTER

SPEECH AND HEARING DEPARTMENT

MASTER'S PROGRAM IN HUMAN COMMUNICATIONS DISORDERS SCIENCES

FIELD OF INTEREST: HEARING - CLINICAL AUDIOLOGY

**QUESTIONNAIRE**

Evaluation Date: ______________

Name: _____________________________________ DB: ______________ Age: _______

Address: _____________________________________

Telephone: ( ) __________ Occupation: ______________ Gender: ( ) F ( ) M

1. Background associated with the initial interview at the time of selection and fitting of the hearing aid (s): __________________________________________________________________________________________________________________________
2. For how long have you been wearing a hearing aid? _____________________________
3. What is the type of fitting? ( ) monoaural ( ) binaural
4. What is the make and model of the hearing aid (s)? _______________________
5. Wear the hearing aid full time? ( ) yes ( ) no ___________
6. Had any battery-related difficulty? ___________________________________________________________________
7. Had any type of difficulty associated with the ear mold (s)? ____________________________________________________
8. Had any difficulty associated with the settings of the hearing aid (s)? _______________________________________________________________
9. If the hearing aid (s) requires any fixing, do you authorize it and would you be able to afford it? ________________________________________
10. If the ear mold (s) needs to be remade, would you be able to afford it? _________________
11. Is there any place in your town to buy hearing aid batteries? ( ) yes ( ) no
12. Is there an otorhinolaryngologist in your city for consultation? ( ) yes ( ) no
13. Is there an audiologist in your city for consultation? ( ) yes ( ) no
14. Is there any doubt in relation to battery use and handling, ear mold and/or hearing device?

hearing aid use, because clinical experience has shown that this is a reasonable time span for the person to get used to wearing the hearing aid, when it is possible to assess the real results of the intervention, since the benefits provided by the amplification do not come immediately^5^,^7^.

Integral use of hearing aid was considered when the user reported that he/she used the hearing aid the entire time, in all activities of his/her daily lives; partial use when it was used everyday, but only during part of the time; and sometimes; when it was used in specific or eventual situations; alternate when it was used sometimes on the right and sometimes on the left ear; and no use, when the person reported not using it because of technical problems or the need for adjustment. Of the 31 individuals, 18 used it full time, while the other 13 used it irregularly; 3 used it partially, 3 in an alternate fashion; 4 used it sometimes and 4 did not use it at all.

The evaluations carried out during the selection and initial fitting process for hearing aids were not considered because they were not part of the goal of this study.

The first part of this study was an interview with topics prepared by the examiner, related to the experience and use of hearing aids, complaints related to the use and handling of batteries, ear molds, amplification characteristics and support, as well as access to specialists of the region (Annex I). By the same token, in this first time, we checked the technical conditions of the hearing aids.

Depending on the needs of each case, we adjusted the amplification characteristics and, in the case in which the problem could not be solved in the first session, new appointments were scheduled.

## RESULTS

In Chart 1 we list the complaints and observations obtained in the post-fitting period, regarding the use and handling of batteries and ear molds or capsules (intracanal devices) used by the 31 users of hearing aids in this study.

Chart 2 shows the complaints and observations of the 31 individuals regarding sound amplification characteristics in the post-fitting period related to the hearing aids.

Chart 3 shows the procedure carried out in the post-fitting period according to each complaint or problem related to the amplification characteristics and/or the device circuit of the 31 individuals in this study.

**Chart 1:** List of complaints and observations as to the use and handling of batteries and ear molds or ear devices (N = 31).

Individuals					Complaints and Observations					
		Batteries				Ear molds of Capsules				
	No complaints	No access	No resources	No complaints	Cleaning	Handling	Discomfort	Wax filter ruptured or obstructed	Damaged molds or capsules	External feedback
1	X					X	X			
2	X							X		
3	X				X					
4	X					X	X			
5	X			X						
6	X			X						
7	X			X						
8	X					X				
9	X			X						
10	X			X						
11		X						X		
12	X							X		
13		X		X						
14	X			X						
15			X						X	
16	X								X	
17	X			X						
18	X								X	
19	X						X			
20	X							X		
21	X									X
22	X			X						
23	X			X						
24	X					X				
25	X			X						
26	X							X		
27			X				X			
28	X								X	
29	X								X	
30	X			X						
31	X			X

**Chart 2:** Association between hearing aid wearing and the complaints and observations as to amplification characteristics (N = 31).

						Amplification characteristics						
Individuals	Wear	No complaints	High intensity	Low intensity	Maximum volume	Headaches	Discomfort	Difficulties in noise	Hears but does not understands	Can not hear from a distance	Hearing aid not working	Distorted sound
1	Has not worn it for more than 14 months	X										
2	Has not worn it for six months								X			
3	Full time									X		
4	Sometimes		X			X						
5	Full time	X										
6	Full time	X										
7	Full time		X			X						
8	Alternate use of haring aids						X					
9	Full time	X										
10	Alternate use of haring aids						X					
11	Has not worn it for six months		X			X					X	
12	Full time			X				X			X	
13	Full time	X										
14	Partial (does not use at work)						X					
15	Full time			X								
16	Full time		X	X	X							
17	Full time			X								
18	Full time	X										
19	Sometimes (four days per week)						X					
20	Has not worn for 11 months										X	X
21	Partial (4 hours per day)			X								X
22	Full time		X									
23	Full time			X	X						X	
24	Full time	X										
25	Full time	X										
26	Sometimes (watch TV and attend classes)		X			X	X				X	
27	Sometimes (three days per week and at alternate days) and alternate	X										
28	Sometimes (to talk)						X					
29	Full time							X				
30	Full time	X										
31	Full time										X	X

**Chart 3:** Procedure according to the complaint or statement of the problem after fitting (N = 31).

Individuals	Hearing aid return	New settings	Refitting	Sent for fixing	Instructions
1	X				X
2	X		X		X
3					X
4		X			X
5					X
6					X
7		X			X
8					X
9					X
10					X
11			X	X	X
12			X	X	X
13					X
14					X
15		X			X
16					X
17		X			X
18					X
19		X			X
20			X	X	X
21				X	X
22		X			X
23				X	X
24					X
25					X
26					X
27					X
28		X	X	X	X
29					X
30					X
31		X	X	X	X

## DISCUSSION

For the patient who could use sound amplification, the auditory prosthesis although representing only one of the components, plays a fundamental role in the auditory rehabilitation process, providing to the hearing impaired an acoustic environment similar to the original environment. Notwithstanding, only the user of the hearing aid can define which were the difficulties encountered with the systematic use of sound amplification during the rehabilitation process. For that reason, we interviewed patients with the goal of investigating the aspects related to the use and handling of batteries, ear molds or capsules, and also in relation to the amplification characteristics.

As far as the batteries are concerned, the users were asked whether they had any difficulty in handling or accessing them. We could notice that 87.10% (27) of them did not have any complaints or doubt in relation to the batteries. Nonetheless, 6.45% (2) said they could not afford them, and one of them said he did not use the aid in full time in order to save batteries; and 6.45% (2) of them had difficulties to buy batteries because they were not sold in his city (Chart 1).

According to the report of some interviewees, the batteries were purchased in other cities of the region, and in some cases they were supplied by the Health Secretariat of the Municipality. One must consider factors like this, in other words, the monetary costs involved with the rehabilitation process, even in the cases fitted by the Public Health Care System - Sistema Único de Saúde (SUS), when technical support and maintenance often times are paid for by the user, interfering in different aspects such as: acceptance, performance, benefit and satisfaction with the use of hearing aid^8^.

Regarding the use and handling of ear molds or capsules, 12.40% (4) of the users reported handling difficulties. The discomfort associated with fitting the ear mold to the pinna or the capsule in the external acoustic meatus was reported by 12.40% (4) of them. Only 3.23% (1) of the individuals reported they did not know how to clean the molds (Chart 1).

Moreover, as we inspected the molds and capsules, we noticed that in 16.13% (5) of the cases, the wax filter was ruptured or obstructed, and only one of them made a regular use of the device, two did not do it for over six months, one for more than 11 years, and another wore only to watch TV or attend classes. We also noticed that 12.90% (4) of the cases had tube in the rigid molds or were cracked and 3.22% (1) had a broken capsule, and only one used the hearing aid irregularly. We noticed the presence of noise or feedback in only 3.22% (1) of the cases (Chart 1).

Although all individuals were educated and instructed during the fitting process, regarding the use and handling of batteries, molds and hearing aids, as well as long term follow up, we found some problems that were not properly solved. It is believed that although satisfaction and use are also associated with the quality of service that the user receives, intellectual and social level, as well as the emotional aspects and motivation involved at the time of fitting, were some of the factors that remained throughout the rehabilitation process.

No complaints and/or observations associated with the amplification characteristics and hearing aid circuit for 10 (35.26%) of the 31 individuals interviewed in the post-fitting period. Nonetheless, one of them said he was not wearing the hearing aid for 14 months, because he did not feel any benefit from it and the mold hurt him, then he decided to return it. Nine others used it full time and one of them used it three times a week, alternatively (Chart 2).

The remaining, 64.74% (21) of the users presented one or more complaints and/or problems associated with the hearing aid devices, namely 28.57% (6) reported strong intensity; 28.57% (6) weak intensity, and two used it at maximum volume; 28.57% (6) discomfort was most of the times associated with binaural use; 19.05% (4) frequent headaches; 9.52% (2) with difficulties caused by the noise; 4.76% (1) could hear, but did not understand; and 4.76% (1) could not hear from far sources. Of these individuals, 10 remained using their hearing aids in full time and the other 11 used them irregularly (Chart 2).

We stress that 33.33% (7) of the individuals had their hearing aids not working or with distorted sound. Despite these occurrences, three of them remained using them, two used them irregularly, one had not used it for more than 6 months and the other hadn't used it for more than 11 months.

Although many of the users had some complaint and/or technical problem, there was not direct association with the time or frequency of use, by the same token, none of them sought help for their problems, justifying it with aspects such as: financial difficulties, accessibility or availability. Nonetheless, we noticed they were not very interested in solving the problems; neither the user nor the municipal health care network, which was often times communicated about the problems found among their users. It is very likely that this is due to the fact that often times communication difficulties remained despite the use of amplification1. By the same token, it is very likely that the perspectives of these users were associated with a curative treatment and not enabling or rehabilitating^9^.

Of the 31 individuals who came to the appointments, 12.90% (4) had neurological issues, three of them had some complaint, and only one of them did not use the hearing aid regularly. Other 6.45% (2) apparently had some depression, used the hearing aid wrongly, both with some type of complaint. We also noticed that 12.90% (4) of the users strongly denied their hearing impairment, and only one of them wore his hearing device the entire time.

In light of the problems found, it was necessary to change amplification characteristics in 25.80% (8) of the cases. Moreover, nine devices needed to be fixed 22.58% (7), and of them, only five were re-fitted during the investigation period. By the same token, another one was re-fitted because it was not used in the last months before the study (Chart 3).

To begin with, these users had restricted access, most of the time needing the aid of the Municipal Secretaries of Health, 76 appointments were scheduled and 46 of them actually happened. With this, we can see the enormous difficulty of bringing awareness, considering the user and the health care network as to the importance of follow up and advice throughout the fitting process. Thus, studies like the present one must be constantly held, not only with a scientific goal, but also to bring awareness to the system and its users that the preferential health care model is not curative, but rather of full health care, in such a way that the user himself would be responsible for his own health^9^, and also responsible for the success of his rehabilitation.

Another issue of major relevance considered by the members of this study are the monetary costs involved with the process, because although they were adapted by means of a consortium for providing hearing aids through the Public Health Care System (SUS), it is also necessary to have resources for providing technical support and maintenance for these devices, just as there is the need for transportation availability to the health care facility for follow up. These are some aspects that also justify the delay in seeking to solve the complaints and/or problems found.

It is known that the audiologic rehabilitation results depend on the active desire by the user, however, the perceived benefit of using amplification is directly associated with the user's expectations^8^, accepting one's hearing loss, the motivation to obtain help, communication needs, financial concerns and social and intellectual level of the hearing impaired2, psychological and social aspects and costs involved in the fitting process, care and maintenance8. It still depends on the problems found during the fitting process and also on the difficulties that still remained with the use of sound amplification and the other ones that stem as a consequence.

These aspects must receive special attention during hearing aid fitting process, especially during the stage of advice and guiding, which must be revised also after fitting, when there are less variables involved, because then better results may be seen and validated along the process^10^.

## CONCLUSION

As we analyze qualitatively the results of the fitting process in this group, we can conclude that:

1. difficulties in relation to the molds or ear capsules and the amplification characteristics were reported by most individuals;
2. the results of this intervention are directly associated, especially to the expectations, communication needs and financial concerns of the user himself, and also to the support provided to this group of patients by the municipal health care network.
